# The relational modeling of hierarchical data in biodiversity databases

**DOI:** 10.1093/database/baae107

**Published:** 2024-10-10

**Authors:** Petr Novotný, Jan Wild

**Affiliations:** Department of Biology Education & Herbarium collections (PRC), Faculty of Science, Charles University, Viničná 7, Praha 128 00, Czech Republic; Institute of Botany of the Czech Academy of Sciences, Zámek 1, Průhonice 252 43, Czech Republic

## Abstract

The unifying element of all biodiversity data is the issue of taxon hierarchy modeling. We compared 25 existing databases in terms of handling taxa hierarchy and presentation of this data. We used documentation or demo installations of databases as a source of information and next in line was the analysis of structures using R packages provided by inspected platforms. If neither of these was available, we used the public interface of individual databases. For almost half (12) of the databases analyzed, we did not find any formalized taxa hierarchy data structure, providing only biological information about taxon membership in higher ranks, which is not fully formalizable and thus not generally usable. The least effective Adjacency List model (storing parentId of a taxon) dominates among the remaining providers. This study demonstrates the lack of attention paid by current biodiversity databases to modeling taxon hierarchy, particularly to making it available to researchers in the form of a hierarchical data structure within the data provided. For biodiversity relational databases, the Closure Table type is the most suitable of the known data models, which also corresponds to the ontology concept. However, its use is rather sporadic within the biodiversity databases ecosystem.

## Introduction

There is a rich discussion around the concept of a taxon in biodiversity databases, focused on the “suitability/correctness of the database model.” In our text, we want to take a step aside from the dominant conceptual debate, i.e. whether indeed from a biologist’s perspective taxon concepts “are often incorrectly modeled in biodiversity databases” [1], and instead look at how efficiently, technically useful, and “usable” hierarchical structures are modeled in them. We are motivated by the insight that the software making data available is similarly critical to the data themselves [2], and the retroactive impact of databases on the user community is rising in importance. The sum of their conceptual and technical properties has a major impact on how researchers conduct and communicate their research [3].

However, let us first recall a source of tension in the field of biodiversity data models, i.e., in our opinion, we make little distinction between the various aspects of such an assessment. At the conceptual level, it is crucial to distinguish whether biodiversity databases are expected to model natural reality or the taxonomy methodology. If a given database is supposed to model “natural reality,” then conceptually it is based on the idea that the naturalness of e.g. vascular plant taxa—their unique origin and coherence through time—is thought to exist independently of human classifications [4] and is thus permissible to exist only single one concept of a taxon.

A very different case arises if the database is to model the “processes of taxonomy,” i.e. the multidimensional universe of relationships between publications, names, herbarium specimens, and past and present meanings (more detailed in [4]). The appropriateness of the database model is incomparable in these cases—they are different models of different objects. Grenié *et al*. [5] aptly use a typology of biodiversity databases when they talk about taxonomic breadth and spatial scale. These features, as such cases that are not globally oriented databases, are perhaps not accidental imperfections, but the foundation of a different conceptual focus which very likely requires a different database model.

Therefore, the adequacy of a database model needs to be viewed from multiple perspectives—whether it is conceptual consistency with the modeled reality, consistency with the conventions of the professional community, or the adequacy of the abstraction used to the practical needs of users.

In the technical sense, a model is the medium to record the structure of an object in a more or less abstract way, following predefined and documented rules [6]. Hierarchical structures are a very typical and important structure in the field of bioinformatics [7]. Whether a biodiversity database operates with a hierarchy of names (i.e. biological database designs ignoring complexity by using only taxon names to identify taxa [8]), taxon concepts/potential taxa (6 and following), or any other concept of biodiversity, the unifying element is the presence of entities that are arranged in a hierarchical or tree structure [9]. This type of structure is found in many other areas of human concern and as such is well studied.

The decision of the appropriate model for hierarchical data significantly affects not only the internal database processes but also the ergonomics of data analysis and processing by users. We have therefore focused on the analysis of modeling tree structures in relational biodiversity databases, which are currently used by most institutions to facilitate the management and accessibility of biodiversity data [1].

### Description

**Figure 1.**
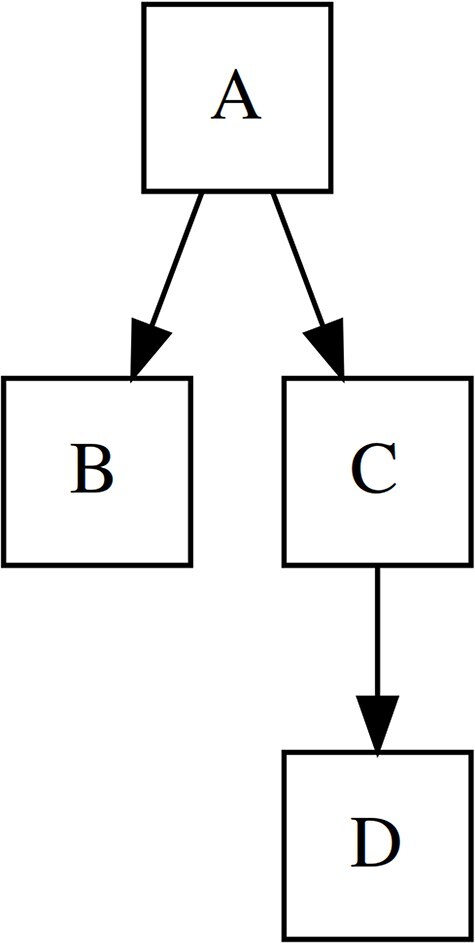
Terminology of tree elements used in this paper. A is a root node, C is a direct parent of D, B is a sibling of C. Nodes C + D represent a branch while nodes B and D are leaves, as they have no children.

The hierarchical organization of data is used very often, e.g., in the computer file system, the territorial-administrative division, or the biological system, but the question of modeling hierarchy in biodiversity databases is still little discussed in the professional discourse, and therefore, in addition to presenting the theoretical aspects, we have set as an objective. In this section, we will use the established terms node, root, parent, child, direct child, sibling, branch, and leaf for a clear description of hierarchical structures (see Fig. 1 for more details).

The graph theory deals with hierarchical data as a directed acyclic graph (DAG), which is a directed graph with no directed cycles. Trees are used for classification purposes, i.e. to provide a unique location of a node in the structure. Trees are a subset of DAGs specific in that a child can only have one parent, so called by another name the uniparental DAG (i.e. they conform to the convention that lichens, e.g., are not classified into both higher taxa, but always into just one fungal parent). At this point, we will narrow the description to tree structures only, but we will return to the issue of multiparental DAGs in the context of biodiversity modeling in the discussion.

**Figure 2.**
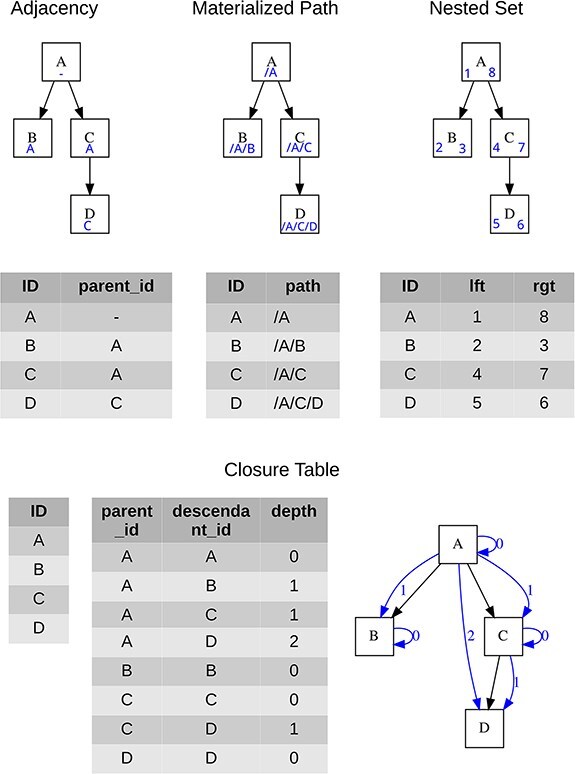
Visualization of the tree and the minimum necessary content of data tables for each data model; blue symbols represent structures used for storing the tree hierarchy.

Four approaches are generally used for storing tree structures in relational databases, see Celko [10] for more details, each of which brings certain advantages, and the specific choice is influenced by the context of use. A brief overview is given in Table 1, while the specific data form for each hierarchical structure model is presented in Fig. 2.

**Table 1.** Overview of relational database tree data modeling approaches with basic characteristics

	Adjacency List	Materialized Path	Nested Set	Closure Table
Basic mechanism	Each node has its parent’s ID stored	Each node has its full hierarchical position stored	The whole tree is double-sided numbered	Each node has a defined link to itself and all its descendants
Minimal count of tables used for objects	1	1	1	2 (hierarchy is separated from objects)
Implementation relevance				
Reading subtree performance	Low	Medium	High	High
Add/move node performance	High	Medium	Low	Medium to low
“ontogeny” capable	No	No	No	Yes
Users’ relevance				
Implicit preservation of sibling nodes order	No	No	Yes	No
Ease of filtering “in Excel”	Low	Medium	High	Low
Ease of editing “in Excel”	High	Medium	Low	Medium

Classification of performance level is only indicative, as it depends on tree size or edited/added node position. The ontogeny capability refers to implicit opportunity in modeling more types of relationships besides the default parent–child relationship. “Users’ relevance” represents the usability of the hierarchy model when data are downloaded and processed in simple data settings, like in a spreadsheet.

#### Adjacency List

It represents an intuitive tree structure model, where the classification is realized in the model employing a recursive relationship between entities of entities. It is generally understandable and easy to maintain even for a layman creating his tree in a spreadsheet. It has been used by historical information model designs for (mostly botanical) databases (like [6, 8, 11, 12]). Its main weaknesses lie in read operations where it requires recursive procedures.

#### Materialized Path

The Materialized Path model stores at each tree node the complete “path” of the node’s ancestors. Although the Materialized Path requires working with strings and regular expressions, it provides more flexibility in working with the tree than the previous model.

#### Nested set

The Nested Set model identifies each node’s position in the tree as stops in a round-trip traversal of the tree starting on the “left” side of the node. The traversal visits each node in the tree twice assigning numbers in the order of visiting, and at both visits. This results in two numbers for each node, which are stored as two attributes.

Although the entire hierarchy is determined by left + right values, for practical use it is useful to add a redundant depth value to the data structure. Attribute depth is dynamically computable, but storing it statically speeds up tree searches and facilitates direct queries, e.g. on direct descendants/parents.

#### Closure Table

The most general tree modeling approach is a Closure Table model. In this model, the relationships are kept in a separate table. It is very important that it can discern different types of relationships, i.e. it can model not only parent–child relationships but also multiparental structures or other relational structures essential for biology. This approach, being similar to ontogenies has the potential to be familiar to biodiversity database users.

In regards to the overall load on the database biodiversity backbone, the most common queries are read-only, and they are often requested at the level of aggregated branches (e.g. species occurrence including subspecies), rather than individual nodes. Parent lookup operations or clustering of branches across a tree are very common and in demand [7, 13].

Therefore, fast read rates at the node and branch level will be an important criterion for selecting a model in terms of internal implementation. The Nested Set and Closure Table models seem to be suitable, which can be indexed very efficiently and therefore searched quickly. Writing a new node or moving an existing one is usually a nonbottleneck operation in biodiversity databases and its computational complexity will play a less important role in model selection. Related to the editing of hierarchies is also the issue of preserving a record of these changes; we will return to this important aspect in the context of biodiversity databases in our discussion.

A common request for trees is also a fixed ordering of the descendants of a given node, e.g. corresponding to the phylogenetic background of taxa [7]. Although it is not difficult to store the order of siblings as metadata to the parent tree, the Nested Set model has the advantage of implicitly containing this information. Thus, if a database export intended for users contains tree structure information, the Nested Set ordering is an advantage.

In Table 1 we also included characteristics that are oriented to the database user, i.e. the usability of individual modeling approaches in a pure user environment, e.g. in an Excel spreadsheet (or simple *dplyr*-style operations in R) that the user downloads from the biodiversity database. Again, we consider the operation of reading and filtering the data as essential; the need to edit the hierarchy is rather theoretical but we present it to emphasize the fact that no modeling strategy is ideal in all its aspects. While in Adjacency List and Materialized Path, the read operation over the tree is at the level of more or less manual work, the two more derived models have much better properties. Some common tasks are demonstrated in Table 2. A complication for the Closure Table is the separation of the data and hierarchical structure into two independent tables which requires a join between the two tables.

**Table 2.** Common filtering tasks on tree modeled with Nested Set and Closure Table

Filtering task	Nested Set model^a^	Closure Table model
All parents	lft < node.lft && rgt > node.rgt	descendant_id == node.id && depth > 0
Direct parent	lft < node.lft && rgt > node.rgt && depth == node.depth—1	descendant_id == node.id && depth =1
Subtree	lft > node.lft && rgt < node.rgt	parent_id == node.id && depth > 0
Subtree including subtree-root	lft ≥ node.lft && rgt ≤ node.rgt	parent_id == node.id
Direct childern	lft > node.lft && rgt < node.rgt && depth == node.depth + 1	parent_id == node.id && depth = 1
Is leaf (have no children)	lft == rgt-1	NOT EXISTS (parent_id == node.id && depth > 0)
Is root	depth == 0	NOT EXISTS (descendant_id == node.id && depth > 0)

a Ascending sort by node.lft provides hierarchically ordered results including the order of siblings. “Node” represents the node of the structure we are referring to, “node.lft” is “lft” value of this node. Characters “&&” represent logical conjunction, and characters “==“represent equality. In the case of Closure Table, the “node” column is stored in one table and all remaining necessary fields are in another table.

The possibility of alternative classifications, which is required for databases that model taxonomic processes [7, 8], is not implicit in any of these models. Thus, restricting to a single classification or allowing alternative classification schemes is a related decision at the level of implementation detail of a particular application, and the principled approaches to hierarchy modeling described remain identical.

Biology has its own, traditional way of modeling real biodiversity, biological systems, and taxonomy. In terms of the above classification, it could be seen as an “inconsistent implementation” of the Materialized Path model (especially for ranks genus and lower). However, the taxonomic model of biodiversity is difficult to formalize [9] due to, among other things, the concept of ranks, so although it is a necessary and fundamental model for the intellectual work of biologists, it is inappropriate for use in relational databases. To store a tree structure, one should use one of the approaches presented above and treat “biological concretization” such as names or ranks of biological entities as metadata of the nodes of this structure.

## Materials and methods

To show the variability of approaches used in current solutions and to demonstrate the importance of the choice of model type in the context of their users, we analyzed a set of established databases to show which hierarchical structure modeling approaches today’s relational biodiversity databases use.

To objectify the selection of the included platforms, we used 12 well-established biodiversity databases as a basis according to the paper of Feng *et al*. [14], to which we added the herbarium systems reported by Gadelha Jr *et al*. [1]. We added four major global checklists of vascular plant names according to Schellenberger Costa *et al*. [15]. The last group of nine software platforms consists of local databases that we use ourselves or came across during our survey.

To determine how the tree structure is modeled in the database, we used only publicly available sources in the following order: the preferred source was the documentation or demo installations within which the database schema can be addressed directly. This was rather sporadic, i.e. if we were unsuccessful, we followed the second approach by using the R package used by the platform. In the case where neither of the previous methods led to the identification of any of the formalized hierarchy entries, we then proceeded to inspect the web interface of the public application and search for possible sources of information such as taxon exports.

If we were unable to download any export or to find more details in the documentation and we were only able to find information about the hierarchy by “reading on the web pages,” it is rated as “not identified.” There was also a case where it was possible to examine the relational structure of the database and confirm the absence of a hierarchical model of taxa, in which case is rated as “none.”

If we encountered only a rank-based hierarchy within a given platform, i.e. a relationally informalized model, is marked as “rank/biological.”

## Results

In total, we analyzed 25 biodiversity software whose summary is given in Table 3. For about half of them (12 platforms) we did not find a formalized data structure to represent the hierarchy. For six cases in this group, the structure was represented using a biological system (i.e. columns for individual ranks); for five cases, we were unable to determine from publicly available sources whether and what type of hierarchy modeling they used, mainly because they do not offer a public interface for downloading taxon lists or similar functionality. For one (BRAHMS) we classified as “none” based on the database schema, a situation where it does not store the taxon hierarchy at all.

**Table 3.** Overview of examined biodiversity software and navigation path leading to the approximation of the model used

Database	Reference	Medium used for inspection	Examined link/operation	Tree representation
“Big12” sensu Feng *et al*. [14]				
Atlas of Living Australia	[17]	Web UI	https://bie.ala.org.au/species/https://id.biodiversity.org.au/taxon/apni/51412617	Adjacency List
Botanical Information and Ecology Network	[18]	R code	BIEN::BIEN_phylogeny_conservative(“Caryophyllales”)	Object of class ape::phylo, i.e. Adjacency List
Biodiversity Information Serving Our Nation	Not inspected, moved to GBIF			
eBird	[19]	Documentation	https://www.birds.cornell.edu/clementschecklist/wp-content/uploads/2022/10/ebird_taxonomy_v2022.xlsx	Rank/biological
Encyclopedia of Life	[20]	Documentation		Neo4j graph database
Global Biodiversity Information Facility	[21]	GBIF Backbone Taxonomy download archive—CSV file	https://hosted-datasets.gbif.org/datasets/backbone/current/	Adjacency List
Global Inventory of Floras and Traits	[22]	R code	GIFT::GIFT_taxonomy()	Nested Set
Integrated Digitized Biocollections	[23]	Web UI	https://www.idigbio.org/portal/search	dwc:higherClassification darwin Core, i.e. Materialized Path
iNaturalist	[24]	Web UI/documentation	Not identified	
Map of Life	[25]	Web UI	https://mol.org/regions/?regiontype=point&location=49.75105923257857,15.3416869909362	rank/biological
Plant Trait Database	[26]	Web UI/documentation	Not identified	
VertNet	[27]	Web UI	http://portal.vertnet.org/search?q=salamander	dwc:higherClassification darwin Core, i.e. Materialized Path
Global checklists of vascular plants				
Leipzig Catalogue of Vascular Plants	[28]	R code	lcvplants::lcvp_group_search(“Caryophyllaceae”, search_by = “Family”)	Rank/biological
World Checklist of Vascular Plants	[29]	Raw data download	http://sftp.kew.org/pub/data-repositories/WCVP	Rank/biological
World Flora Online	[30]	Raw data download	http://www.worldfloraonline.org/downloadData	Rank/biological
WorldPlants	[31]	Web UI	Not identified	
Herbarium systems according Gadelha Jr *et al*. [1]				
BG-BASE	[32]	Documentation	Not identified	
BRAHMS	[33]	Demo database structure	https://herbaria.plants.ox.ac.uk/bol/brahms/support/conifers	None
EMu	[34]	Documentation	Not identified	
Specify	[35]	Documentation	https://www.specifysoftware.org/specify-6-schema/	Adjacency List
Others				
Avibase	[36]	Documentation		Adjacency List
Biologer	[37]	Documentation		Adjacency List
Dyntaxa	[38]	Documentation		Closure Table
Finnish Biodiversity Information Facility	[39]	Web UI	https://laji.fi/en/taxon/list?target=MX.70046&onlyFinnish=true	Rank/biological
Pladias	[40]	Web UI	https://pladias.ibot.cas.cz/query/taxonDetails	Nested Set
TaxonWorks	[41]	Documentation	https://docs.taxonworks.org/develop/Data/models.html#taxon-name-relationship	Closure Table

The remaining sources contained a formalized tree model which was most often Adjacency List [6], the other models (Materialized Path, Nested Set, and Closure Table) had two instances each. Databases that represent the tree structure in the taxon export following the Darwin Core standard, specifically *dwc:higherClassification* in line with the recommendation to provide “values in a list with space vertical bar space (|), with terms in order from the highest taxonomic rank to the lowest” [16], were classified as Materialized Path.

In the Botanical Information and Ecology Network case where the taxon hierarchy was obtained through the R package BIEN, the hierarchy model was available as an object of the class *ape::phylo*. Although, as a phylogeny, it contains data describing partial relationships between nodes (and thus is similar to the Closure Table model), in terms of working with a hierarchical tree it corresponds to the Adjacency List model and we classified it as such.

Encyclopedia of Life has its backbone built not on relational databases but based on Neo4j graph database, i.e. in an environment that models trees quite differently.

## Discussion

As mentioned in the introduction, we can expect the biodiversity database to model natural reality, the processes of systematics, or the resulting taxonomy—the traditional mental model of biologists. Each of these realities presents different conceptual challenges for database development, and the discussion to find the right model can hardly converge to a successful solution without a clearer distinction of the essence. We have chosen the issue of tree structures in relational biodiversity databases because we consider them to be the central structural element that is present in them, regardless of the conceptual differences indicated. Although relational databases have recently gained competing solutions in the form of graph databases that change the view of storing tree structures, the relational approach is currently the most widely used.

In relational software, the choice of the model for the tree structure is crucial for the relevance between the software model and the modeled reality, for the efficiency of the database operation, and is also relevant for the user. The role of the user of a biodiversity database can hardly be overestimated. In line with Gadelha Jr *et al*. [1], this role has the potential to provide users with valuable information, a fundamental component of data quality. Therefore, even when storing data in graph databases, the interest in making available a formalized tree structure remains relevant, as the typical format of data exchange between a database and its professional users is in the form of CSV-type spreadsheet formats, in which graph data must also be reformatted into some relational model for usability.

As an example to describe this problem, we will use the Dyntaxa database for which we have realized how isolated we often look at the individual application layers and the needs of their users; however, this is undoubtedly a common situation in other databases as well. The Dyntaxa documentation provides a detailed description of the relational structure [38, p. 10], so it is clear that it uses the Closure Table model and we have classified it as such in our overview. While exploring the UI of the application and looking for how to obtain a formalized model of the hierarchical structure, we found a dedicated tool for exporting taxa lists, including a so-called “hierarchical” mode (https://www.dyntaxa.se/Export/HierarchicalTaxonList/3000462). Despite the name, however, it only provides users with hierarchy information in the form of biological ranks in the downloaded Excel spreadsheet. Thus, although the database models the relationships between taxa with the modern and well-described Closure Table formalization, it does not provide it to users for some reason.

Undoubtedly, a field biologist needs insight into the data described by the rankings, as this corresponds to the “cognitive pragmatics” of biologists [42], but for “analytic pragmatics” we would expect biodiversity databases to also offer a formalized notation of tree structure, which is needed in almost every data analysis nowadays. A formalized notation of the taxon hierarchy should therefore become part of the requirements for “Next-Generation Collections” [43], as tools that support data usability and machine processing in analyses. Which of the possible models is appropriate is up to the operator’s discretion to choose the one that best suits the intended needs of the database and its users, but in short, it is necessary to have both a taxonomic and formalized notation of the data structure.

We do not want to label appropriate and inappropriate within the four modeling approaches—each of the formalized notations has its own strengths and weaknesses. Still, we nevertheless attempt to discuss relevant properties to biodiversity databases below.

Three models are only applicable to databases targeting classification models that work with uniparental DAGs.

Adjacency List is the most commonly used formalized structure notation we found. Its computational complexity for analyses is counterbalanced by its intuitive understandability for all users. From the point of view of internal database design, we consider it deprecated. In terms of presenting the data to users, we believe that the basic tree manipulations within the more derived models (as shown in Table 2) are, with the provision of some minor hints, easily understandable to nontechnical users. However, it is also relevant to consider the use and convenience of practical users. In the extreme position of our reasoning, one can consider that, independently of the internal implementation within the database model, it would be useful to provide users with complementary, computed hierarchical level models, precisely including the Adjacency List. To our knowledge, no biodiversity database provides data in this way, although it is not a technically challenging solution.

Materialized Path seems nowadays heavy-handed at the database model level. According to our findings, it is not often used in biodiversity databases and its observed occurrence is equivalent to the use of DarwinCore in exports. In such export mode, we find it useful when passing data for individual taxa or selecting unrelated taxa because, unlike other models, it does not require the listing of all parent nodes (higher taxa) and thus does not ballast the multiplication of rows in the output. In this sense, its use in the DarwinCore field *dwc:higherClassification* [16] is logical. At the same time, however, the exports already contain the taxon classification using ranks, from which the Materialized Path can be easily inferred.

The Nested Set model represents an interesting and the most abstract solution for classification tree data (i.e. uniparental DAGs). Its structure is highly searchable at the backend storage, but also when the tree is distributed as an Excel table as shown in Table 2. An ambivalent property of the Nested Set is the implicit ordering of descendants—on the one hand, it is positive that the structure itself preserves the order, on the other hand, there are situations where biologists do not have a phylogenetically based intention of sibling order. In such situations, a fixed order is *de facto* misleading.

At the beginning of our study, we pointed out the need to define precisely which reality should be modeled in the database. If the model is to describe natural reality, it must include direct evolutionary relationships between taxa, but uniparental DAGs are no longer sufficient. To store the linkages of both parents of a given hybrid taxon, the only usable relational formalization is the Closure Table, with capability to model a multiparental DAG. It also allows a formulation of a richer typology of relationships (part of, congruent, overlap) between nodes of the hierarchy than simple parent–child. Since many authors see the global future of biodiversity databases as being in the integration of ontologies [44–46] and Closure Table is capable of fulfilling needs for classification trees as well, we can expect its gradual dominance. Its fundamental property is the necessity to separate hierarchy data (ontology for taxa relationships) from the nodes (taxa) themselves. At the level of data presentation, the two-table structure may appear as a handicap for users working in spreadsheets, but it can also be seen as an easy vehicle to start providing the formalized data and “not scare away” natural scientists. In the same way as we discussed the usefulness of providing users with multiple variants of the formal expression of the tree structure, we offer a positive view of this feature of the Closure Table model. It could be a goal of databases to provide checklists of taxa with their biological classification and columns understandable to all biologists, such as “Class” or “Genus” etc., i.e. as the current most common practice looks like, complemented by a second set/table of formalized, ontology-ready structure notation for analysis purposes.

The computational complexity of editing (i.e. adding, moving, and deleting nodes) varies from model to model; see Table 1 for an indicative comparison. However, another requirement—tracking changes—is crucial compared to other tree structure models. Storing changes and presenting them in a comprehensible way is extremely desirable in the context of taxon hierarchy.

The general approach of write-ahead logging, i.e. a separate record of changes made over the initial tree, is possible for all the models discussed. By keeping each partial change, it is then possible to replicate the individual states and describe the differences between them. However, we see a difficulty in the biological interpretability of this approach. When one taxon is moved to another group, it is not only a change in its classification, but it is also a modification of the substantive nature of the initial and target groups. Creating a comprehensible presentation of tree changes that shows the context of changes in the biological concept of a taxon seems to us almost impossible.

Again, the Closure Table model could provide some solution. If we flagged node relationships as valid and invalidated, we could preserve the history of changes within the hierarchy model itself. When relationships are supplemented with a record of their creation and invalidation by reference to the versioning of the entire tree, a model capable of retrospective presentation of the evolution of the stored hierarchy would then be available.

## Conclusion

We have tried to map the approaches that are used nowadays for modeling hierarchical structures in biodiversity databases and how they are provided to the users. In our opinion, the results show one main conclusion, namely that the formalized tree structure model is an overlooked aspect, at least from the users’ point of view. As a crucial point, we make a plea for database operators to realize that they have two tasks concerning tree structures—to model them effectively in the data backbone (which has undoubtedly been done for decades), but also, this is the critical point, provide formalized tree structure models included in the data exports for users’ utility and efficiency.

Of the identified data models in use, we consider Closure Table to be the most relevant for biological hierarchies in relational databases because of its ability to formalize the various types of relationships defining the hierarchy as well as relationships beyond it, including change tracking. For user interfaces and exports, the Nested Set model appears to be suitable, as it excels in the straightforwardness of searching and sorting within the tree.

### Limits

A clear limitation of our review of the current state of hierarchical structure modeling is the methodology of discovering them. We only looked from the outside without insider knowledge of the internal structure of providers. This approach is usable for inspecting user ergonomics, but it limits the statement of what models are used in the db (see the example of Dyntaxa), as the applications may offer other options to internal users. At the same time, it is necessary to admit at least the hypothetical possibility of using one relational model internally and reconstructing it externally into a second model (e.g. Materialized Path of Darwin Core does not necessarily mean that the database stores it that way, it is just presented that way following the standard). Thus, the overview does not necessarily correctly describe the individual databases, but it allows us to illustrate the situation that led us to this work, i.e. the disparate and unappreciated emphasis on a formalized description of hierarchical data towards the public.

Our search focused only on taxon-related structure, but there are other hierarchies in the databases, such as spatial administrative units or phytogeographic subdivisions, and it would certainly be interesting to compare approaches to modeling them.

Although we have included databases with a global scope in the biological and spatial sense, when writing the text, we have in mind a vascular plant as a model taxon. Therefore, it is possible that, given the complexity of genetic flow mechanisms in prokaryotes or viruses or the differences between the nomenclatural-systematic traditions of particular organismal groups, we committed an oversimplification of the biological essence when considering taxon hierarchy. However, this should not undermine our conclusion regarding the necessity of hierarchical structure formalization of hierarchical structures in the context of biodiversity databases.
